# Hazelnut Skin Waste as a Functional Ingredient to Nutritionally Improve a Classic Shortbread Cookie Recipe

**DOI:** 10.3390/foods12142774

**Published:** 2023-07-21

**Authors:** Lara Costantini, Maria Teresa Frangipane, Romina Molinari, Stefania Garzoli, Riccardo Massantini, Nicolò Merendino

**Affiliations:** 1 Department of Ecological and Biological Sciences (DEB), Tuscia University, Largo dell’Università snc, 01100 Viterbo, Italy; 2 Department for Innovation in Biological, Agro-Food and Forest Systems (DIBAF), University of Tuscia, Via San Camillo de Lellis, 01100 Viterbo, Italy; 3 Department of Chemistry and Technology of Drugs, Sapienza University of Rome, Piazzale Aldo Moro 5, 00185 Rome, Italy; 4 Study Alpine Centre, University of Tuscia, Via Rovigo, 7, 38050 Pieve Tesino, Italy

**Keywords:** hazelnut by-product, hazelnut cuticle, hazelnut pellicle, butter cookie, pastry, functional foods, unsaturated fats, saturated fats, oleic acid, sensory analysis

## Abstract

Hazelnut skin (HS) is a byproduct of hazelnut processing (2.5% of total kernel) and becomes a food waste despite its high content of antioxidants, unsaturated fats, and fibers. Classic shortbread cookies have a large worldwide market, even if their nutritional composition does not meet nutritional guidelines due to the high content of saturated fats. In the present study, after the nutritional evaluation of four different HS varieties, 5% and 10% ratios of HS of the Tonda Gentile Romana variety were integrated into a classic shortbread cookie recipe, proportionally replacing the butter amount with the unsaturated fats naturally present in HS. The 10% HS addition determined a 20% increase in the monounsaturated oleic acid and a 15.7% decrease in the saturated palmitic acid, in addition to a significant ash increase. The sensory analysis revealed higher consumer acceptance of the 5% formulation, with scores comparable to the control. Although the 10% formulation obtained lower scores for consumer acceptance, 35% of the interviewed population said they would purchase it, indicating that this product, beyond the ethical dimension of using a food waste matrix to promote the circular economy, can attract the commercial interest of part of the population.

## 1. Introduction

The worldwide hazelnut production in 2021 was 1,077,117 tons [1]. The hazelnut consists of a hard outer shell that contains a kernel, i.e., the commercial product. Following the rupture of the hard outer shell, hazelnut processing involves kernel roasting and the waste of the hazelnut skin (HS), which represents about 2.5% of the total hazelnut kernel [2]. Therefore, because the HS is removed in most types of hazelnut processing, and considering the latest world quantities of hazelnut production, it is estimated that about 27,000 tons of HS are wasted every year. HS is a food matrix with important nutritional features. It has a total antioxidant capacity 25 times higher than blackberries [3], comprises about 50–70% dietary fiber content which is mainly water-insoluble [4,5], and its lipid fraction, although highly variable between varieties (about 14–40%), is rich in tocopherols and oleic acid [6,7].

Shortbread cookies are pastry goods belonging to bakery products that have a large worldwide market (in Italy, baked goods production reached 1,310,412 tons in 2021) [8] for their organoleptic characteristics such as density and brittleness due to the high amount of total fat and high percentages of saturated fatty acids (SFAs), such as myristic, palmitic, and stearic acids [9]. Due to their SFA content, the nutritional composition of shortbread cookies does not meet nutritional guidelines, because high consumption of SFAs is associated with an increased risk of cardiovascular diseases [10]. For this reason, to make these foods healthier, preparing low-fat cookies using emulsifiers, interesterified shortenings, or some vegetable oils rich in unsaturated fatty acids (UFAs) are some of the strategies adopted in recent years [11,12]. Moreover, shortbread cookies are low in fiber, antioxidants, and minerals; therefore, further changes should be made in the current formulations to improve these qualities and make the recipes more compliant with nutritional guidelines [10]. In shortbread cookies, SFAs (as solid fats) allow the trapping of gases from the leavening agents, producing a fluffy texture, whereas UFAs (as liquid fats) produce a flat cookie with a greasy texture due to fat migration given their liquid state [13]. For these reasons, many efforts must be made to improve the sensory profiles of healthier shortbread cookies to make them accepted by consumers, and this is still a future challenge.

Thus, with the aim of improving the overall nutritional value of shortbread cookies, in the present study, the integration of 5% and 10% ratios of HS in two different experimental cookies was performed and analyzed. To do this, first, proximate composition comparisons of four different HS varieties were conducted to identify the one with the best nutritional characteristics. Next, two isolipidic recipes of shortbread cookies enriched with 5% and 10% HS were formulated by partially replacing the butter amount of the classic recipe with the unsaturated fats naturally present in HS. To evaluate the nutritional characteristics and the lipid profile of shortbread cookies, proximate analysis and fatty acid (FA) analysis were performed on raw HS and experimental cookies. Finally, sensory analysis was also carried out to understand if these recipe changes could be accepted by consumers.

## 2. Materials and Methods

### 2.1. Hazelnut Skins and Chemicals

Hazelnut skins of the Tonda Gentile Romana (HSR), Tonda di Giffoni (HSG), Tonda Gentile delle Langhe (HSL), and Turkish Tombul (HST) varieties were obtained from a local producer (Bionocciola srl, Carbognano, Viterbo, Italy) after roasting hazelnuts at 150 °C for 24 min and under conditions suitable for human consumption. Commercial Italian wheat flour type “00”, as legally defined in the Italian Government Official Bulletin (2001), along with butter, sugar, eggs, baking powder, and vanillin were purchased from a local market. All chemicals were purchased from Sigma-Aldrich (St. Louis, MO, USA) unless otherwise specified.

### 2.2. Proximate Composition

Proximate composition was determined according to standard procedures of AOAC International [14]. In particular, moisture content was determined by heating 1 g of the sample at 102 °C in a static oven until constant weight measurements. Crude protein content (conversion factor, 6.25) was estimated using the Kjeldahl method (AOAC 2001.11) (SpeedDigester K-425 and Distillation Unit K-350, BÜCHI, Labortechnik, AG, Switzerland). Crude fat (AOAC 920.39) was extracted using a Soxhlet Extraction System B-811 (BÜCHI, Labortechnik, AG, Switzerland) with petroleum ether as a solvent. Ash was determined in a muffle furnace at 550 °C for 4 h (AOAC 923.03). Total carbohydrates were determined by the difference (i.e., 100 − (g [protein + fat + ash] in 100 g of DW sample)). The energy value was calculated on fresh weight (FW), using the Atwater factor, as follows:

Energy value (Kcal)=(%Protein× 4)+(%Fat× 9)+(%Carbohydrate× 4).

### 2.3. Experimental Cookies’ Preparation

Mixtures of all the ingredients and hazelnut skin from the Tonda Gentile Romana variety, hereafter HSR, were prepared, with ratios of 5% (hazelnut skin cookie 5%, HSc5%) and 10% (hazelnut skin cookie 10%, HSc10%) of the total ingredient amount. HSR was ground in a coffee grinder before being incorporated into the cookie dough. A classic shortbread cookie recipe was used as a control and named the classic shortbread cookie (CSc). In both the HSc5% and HSc10% recipes, the fat from butter was replaced with the fat of the HSR to obtain three isolipidic (i.e., with the same amount of fats) formulations. All the ingredients were mixed in a mixer for 10 min. All the doughs made were wrapped in polyethylene bags and left to rest at +4 °C for an hour, and then they were manually sheeted to 5 mm thickness and cut by pressing molds onto dough sheets. Baking was carried out in an oven at 170 °C for 20 min. After cooling for 30 min to room temperature, the cookies were ground in a laboratory mill (IKA^®^ A11 basic Analytical mill (IKA^®^-Werke GmbH & CO., KG, Staufen im Breisgau, Germany)). After that, the powders were packed in sealed polypropylene bags and kept at −20 °C for further examination. Cookie powder was used for all the analyses.

### 2.4. Fatty Acid (FA) Profile

FA composition was determined by the gas chromatography/mass spectrometry (GC/MS) technique after lipid extraction and the synthesis of FA methyl esters, performed according to Costantini et al. [15] and Farinon et al. [16]. The analyses were carried out on a gas chromatograph equipped with a flame ionization detector (FID) and directly coupled to a mass spectrometer (MS) (Perkin Elmer Clarus 500 model (Waltham, MA, USA)). The GC oven was fitted with a 60 m × 0.25 mm Restek Stabilwax polar capillary column with a film thickness of 0.5 μm. Helium was used as the carrier gas at 1.0 mL/min constant flow. The injector was set to 280 °C and the programmed temperature was set to 170 °C at a rate of 3 °C/min to 260 °C for 10 min. Then, 2 μL of each sample was injected into the column in splitless mode; the mass spectra were recorded at 70 eV (EI) and scanned in the range of 40–550 *m*/*z*. The ion source and the connection part temperature were at 220 °C. The GC-TIC mass spectra were obtained by the TurboMass data analysis software (Vers. 6.1.0). Electronic integration measurements of peak areas by the FID detector were used to generate quantitative data of all the compounds without the use of an internal standard or correction factors and expressed in percentages; the identification was performed by matching their mass spectra with those stored in the Wiley mass spectra library database.

### 2.5. Sensory Analysis

Sensory evaluation, a technique largely applied to a wide range of foods [17], was adopted to evaluate the acceptability of the experimental cookie samples. The test was carried out with 118 non-trained assessors who were students and employees of Tuscia University (Viterbo, Italy). The recruited people were male and female adults between the ages of 18 and 65 years. Following the tasting, each consumer could fill out an online questionnaire which they could access anonymously by scanning a QR code with their mobile device. The tasting was carried out without knowing the composition of the three experimental biscuits in order not to influence the opinion of the consumer, who was informed about the composition of the product only at the end of the test. Participation was voluntary and anonymous, and each consumer had to declare in the online form that they did not have any food allergies or intolerances and to authorize the researchers to use the anonymous data for experimental purposes before proceeding with the test. The three cookie samples were differently coded as follows: HSc5% as (A), HSc10% as (B), and CSc as (C). They were packed in individual polyethylene bags on which the code of the sample was noted, and they were provided to the consumers in a random order [18]. Water was provided for palate cleansing. An acceptance test was achieved to evaluate overall liking, flavor, aroma, and aroma persistence using a 9-point hedonic scale with “dislike extremely” on the left and “like extremely” on the right. Finally, assessors were asked how much they would presumably purchase each of the three cookies to evaluate purchase intention. Purchase intention was evaluated using a 5-point structured scale ranging from “would certainly buy” to “would certainly not buy”.

### 2.6. Statistical Analysis

The mean and standard deviation (SD) of replicates were calculated for all the analyzed data from the raw material and experimental cookie samples. Statistical analysis was performed with XLSTAT 2023.1.1 (Addinsoft SARL, New York, NY, USA) software using one-way ANOVA. Fisher’s least significant difference test was used to describe statistical differences between means at a *p* < 0.05 significance level. Principal component analysis (PCA) was employed to better understand the influence of sensory descriptors on the overall liking of the cookie samples.

## 3. Results and Discussion

### 3.1. HS Varieties’ Proximate Composition

In the present paper, for the first time, a proximate composition comparison of four different hazelnut varieties—three originating from Italy, i.e., Tonda Gentile Romana (HSR), Tonda di Giffoni (HSG), and Tonda Gentile delle Langhe (HSL), and one from Turkey, i.e., Turkish Tombul (HST)—was performed, and the results are shown in Table 1.

The data showed higher moisture content for HSR, followed by HST, HSL, and HSG samples with progressively lower values, probably due to slightly different environmental humidity levels at the time of storage by the producer. Regardless, the moisture content was comparable, and in some cases lower, than the moisture content found in the literature (4.1–11.89%) [6,7,19]. The HSR variety had the highest protein content, followed by HSG, HST, and finally HSL with the statistically lowest value. The higher value found for HSR (~9%) in comparison to HSG confirmed our previous data [19], and the protein values found for HST agreed with data found in the literature (~8%) [6]. Other authors found higher protein value for HSL (~10%) [7], but, as mentioned above, no comparison in the literature includes all the varieties analyzed here. The highest value of fat was found in the HSG variety, followed by HSR, HSL, and lastly HST. Although our data for HSR fat content are in accordance with our previous data (~26%) [19], different values were found for HSG, HSL, and HST. However, it should be considered that many variable results are found in the literature for total lipid content [9,20], probably due to geographical origin, climatic factors, and different methods of cultivation. The highest ash values were found in the HSR variety, followed by the HSG and HSL varieties, with HST having the lowest value, and these values are comparable to those found in the literature (2–3%) [6,19,20]. The carbohydrates, determined by difference, appear to be highest in the HST variety, followed by the HSL and HSG varieties, and lastly the HSR variety with the lowest value. The lowest energy values are related first to the HST variety, followed by the HSR variety, while the HSL and HSG varieties present the highest values. All the differences are statistically significant between varieties (Table 1).

### 3.2. Formulation of Isolipidic Experimental Cookie Recipes

Based on the nutritional evaluation of the four hazelnut skin varieties, it was possible to identify HSR as the best variety for protein and ash contents, and with appreciable fat amounts (Table 1). Shortbread cookies have a large worldwide market, even if their nutritional composition does not meet nutritional guidelines due to the high content of saturated fats. For this reason, making these foods healthier, and at the same time, acceptable to consumers, is a future challenge.

**Figure 1:**
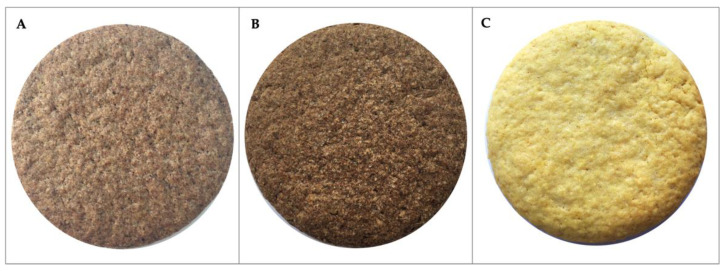
Experimental cookies. (**A**) HSc5%, hazelnut skin cookie 5%; (**B**) HSc10%, hazelnut skin cookie 10%; (**C**) CSc, control cookie.

For all the reasons above, in the present paper, HSR was integrated into two different shortbread cookie recipes in percentages of 5% (HSc5%) and 10% (HSc10%). As a control, a classic shortbread cookie recipe was used, with the percentages of ingredients indicated in Table 2. The butter amount used in the recipe was slightly decreased from the average of the quantities found in the literature [9,21,22,23,24]. With the aim to decrease the saturated fats in the classic recipe by replacing them with the unsaturated fats of HSR, the butter amount was proportionally decreased in HSc5% and HSc10% based on the fat content identified in HSR (Table 1), and considering the amount of HSR used in the experimental recipes (i.e., 5% and 10% HSR) (Table 2). The resulting experimental cookies are shown in Figure 1.

### 3.3. Nutritional Evaluation of Experimental Cookies

Following the formulation, a nutritional evaluation of the three experimental cookies was carried out, and the results are shown in Table 3. The data confirmed the correct dosage of the ingredients to obtain isolipidic formulations of the two types of experimental cookies, HSc5% and HSc10%, in relation to the control CSc. Indeed, no statistically significant differences were found in fat content among the samples. This approach helps to reduce the amount of fat, considering the lipids naturally present in hazelnut skin, and avoid progressive increases in fat and calories, proportional to the addition of hazelnut skin. Indeed, although in a previous paper, hazelnut skin of the Turkish Tombul variety was used in cookie formulations with percentages from 10% to 25%, the amount of butter in the recipes was kept constant among formulations, and the proximate composition was not analyzed; therefore, the total fat and calorie contents are not known [25]. As expected, the HSR addition determined a significant ash increase in both experimental formulations, HSc5% and HSc10%. The significant increase in ash corresponded to an increase in the probably healthy inorganic mineral component, especially considering that no salt was added to the recipe of the experimental cookies. Conversely, the protein content did not change for HSc5% and slightly decreased for HSc10%, maintaining comparable values. Although statistically significant values were recorded for the moisture content, they are very similar to each other. Similarly, for the energy content, some variations were found, but fiber analysis appears necessary to confirm or deny these data (Table 3).

### 3.4. Fatty Acid Content of Experimental Cookies

Since quantitative lipidic analysis confirmed no differences between the experimental samples, qualitative fatty acid analysis was carried out to investigate the type of fatty acids that HSR can contribute to the two experimental formulations; there is no evidence in the literature of the lipid profile of HS-fortified foods. The data found for HSR showed similar amounts of palmitic acid (C16:0, 6.60%), stearic acid (C18:0), and oleic acid (C18:1n9) to data present in the literature [26,27]. These data showed and confirmed the high content of unsaturated fatty acids (UFAs) due to the presence of the monounsaturated oleic acid in comparison to the saturated fatty acids (SFAs) palmitic and stearic acid. Conversely, here, for the first time, the presence of trans-fatty acids (TFAs), i.e., linolelaidic acid (C18:2 n6,9 all trans), was found in hazelnut skin. This can be due to the heat-induced cis-trans isomerization of UFAs due to the high roasting temperature. The ratio between UFAs and SFAs and between UFAs and TFAs is always high and positive, in favor of UFAs (i.e., 9.53 and 7.29, respectively; Table 4). Here, for the first time, the fatty acid profile of HS-enriched foods (i.e., shortbread cookies) was analyzed. The incorporation of 5% and 10% of HSR in the classic shortbread cookie recipe determined a significant decrease in palmitic acid (C16:0) in HSc10% (33.75%) in comparison to CSc (40.05%); a decrease, although not significant, in stearic acid (C18:0) among the three experimental cookies; and a significant increase in oleic acid (C18:1 n9) in HSc10% (34.35%) in comparison to CSc (28.65%). For these differences, the UFA/SFA ratio rose from 0.45 in CSc to 0.60 in HSc10%. However, these significant differences were not found in the HSc5% formulation, where 5% HSR did not improve the lipid profile. Moreover, due to the increasing incorporation of HSR, higher and more significant linolelaidic acid content was found in HSc10% (5.75%) compared to CSc (4.45%). However, it should be noted that the UFA/TFA ratio decreased only slightly among the three experimental cookies and was higher in CSc (6.65) and lower in HSc10% (6.17). Moreover, the TFA content in the experimental cookies is much lower than the World Health Organization (WHO) indication. Indeed, WHO suggested that the intake of TFAs should not outweigh 1% of total daily energy intake, and here, considering a serving size of 30 g, the energy intake from TFA in HSc10% would be 3.3 kcal (0.16% of a 2000 kcal diet) [10]. Further efforts will have to be made to decrease the formation of TFAs during HS processing, acting on roasting temperatures and the methods of separating HS from the kernel.

### 3.5. Sensory Analysis

To understand if the nutritional improvement of the experimental cookies could go hand in hand with consumer acceptability, a sensory analysis with 118 non-trained assessors was carried out, and the results are shown below. The sensory descriptors and the overall liking, reported in Table 5, showed that “Overall liking” and “Aroma” demonstrated a significant difference (*p* < 0.05) between the three samples of the experimental cookies. The “Overall liking” of sample HSc10% had the lowest score (6.20), while sample CSc was the preferred one (7.68) and sample HSc5% earned a middle score of 7.31. The “Aroma” parameter of sample CSc achieved the best score (7.80), but the HSc5% cookie had a high and comparable score (7.59). The findings demonstrate that for the descriptors “Flavor” and “Aroma persistence”, HSc5% and CSc samples did not show any significant difference (*p* ≥ 0.05). Both types were highly accepted by consumers. This confirmed that cookies enriched with 5% of hazelnut skin (HSc5%) received positive scores comparable to those of the control, CSc, and they were better accepted in the sensory test in comparison to cookies enriched with 10% of HSR (HSc10%) (Table 5).

**Figure 2:**
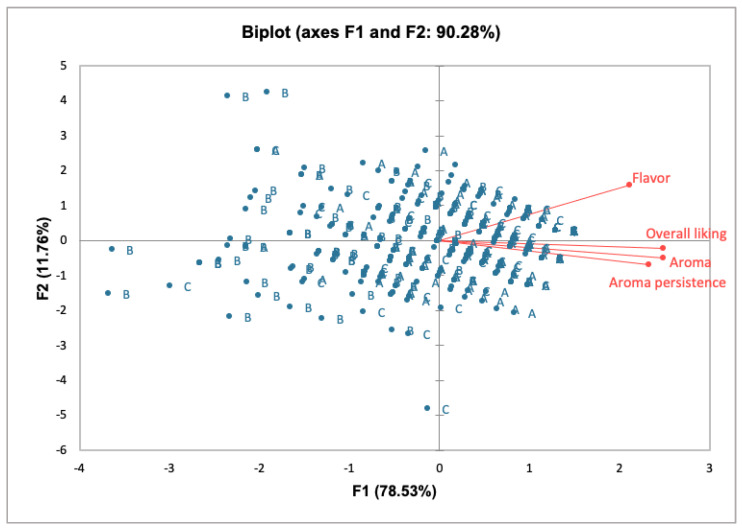
PCA loading plot showing the multivariate variation between cookie samples in terms of sensory descriptors and overall liking. (A) HSc5%, hazelnut skin cookie 5%; (B) HSc10%, hazelnut skin cookie 10%; (C) CSc, control cookie.

To better understand the influence of sensory descriptors on the overall liking of the cookie samples, PCA was carried out. As shown in Figure 2, the first two components together explained 90.28% of the total variance (F1 78.53% and F2 11.76%). Figure 2 reports the consumer preference map associated with the PCA score plot of the data. In this case, the acceptability of the cookie samples in terms of “Flavor”, “Aroma”, and “Aroma persistence” as well as “Overall liking” was oriented toward HSc5% and CSc, whereas the HSc10% cookie sample was the least preferred by consumers. PCA allowed separating samples according to cookie composition along the F1, with HSc5% and CSc samples on the positive side of the F1 and HSc10% on the negative side. The consumers preferred HSc5% and CSc samples for “Aroma”, “Aroma persistence”, and “Overall liking”. Also, regarding “Flavor”, the main preference was observed for HSc5% and CSc cookie samples. The PCA biplot revealed a clustering of the HSc10% sample in the third and fourth quadrants of the plot. Meanwhile, HSc5% and CSc samples were mainly located in the first and second quadrants. This grouping accounted for 90.28% of the total variance in the data set and has been demonstrated to be very useful in interpreting the effect of the different hazelnut skin ratios in cookies on consumer sensory acceptance.

**Figure 3:**
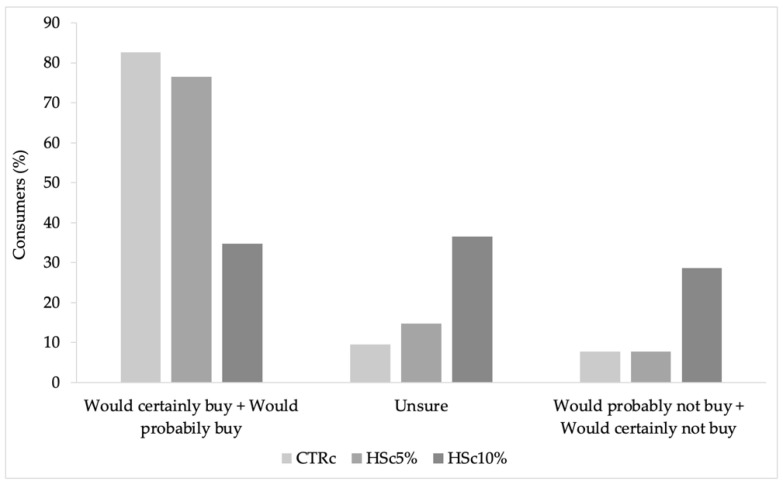
Purchase intention of experimental cookies. CSc: control cookie; HSc5%: hazelnut skin cookie 5%; HSc10%: hazelnut skin cookie 10%.

A purchase intention test was also conducted. It can be seen in Figure 3 that CSc received 82.6% of the evaluations between “would certainly buy” and “would probably buy”, followed by HSc5%, with 76.52% of the evaluations between “would certainly buy” and “would probably buy”, ranking second in consumer choice. These results corroborate the results of the sensory test, where CSc had the highest acceptance mean of overall liking (7.68), followed by HSc5% with 7.31. Regarding HSc10%, consumers were proportionally divided among “would certainly buy” and “would probably buy” with 34.78%, “unsure” with 36.52%, and “would probably not buy” and “would certainly not buy” with 28.7% of the judgments, collecting the lowest percentages for the three types of responses. This result may be associated with the lowest mean (*p* < 0.05) presented by sample HSc10% in all considered attributes (Table 5). Meanwhile, samples HSc5% and CSc had the same percentages of judgments (7.82%) for the answers “would probably not buy” and “would certainly not buy” (Figure 2).

Similar results were also found in the literature for yogurts and vanilla ice cream enriched with roasted HS, and in all these studies, the low acceptance of the HS-enriched foods was explained by the HS fiber content [5,28]. It also seemed that the increase in the HS ratio was proportional to the negative flavor score in the samples. For example, for HS enrichment of 1.5%, 3%, and 4.5% in vanilla ice cream, only the 1.5% ratio was like the control [28]. Similarly, among the 3% and 6% HS enrichment of yogurts, only the 3% enrichment was like the control in terms of flavor score, while for the 6% formulation, the score was halved [5]. Conversely, in the paper of Velioglu et al., the addition of 4%, 6%, 8%, and 10% ratios of HS in cookies led to a growing score in consumer acceptability, but this may be related to the fact that the amount of butter between formulations was unchanged, and the replacement of flour with HS resulted in an increasing amount of total fat between the formulations, which improved their taste and thus their consumer acceptability [25]. In light of these considerations, the results obtained here for HSc5% are better considering the higher HSR percentage employed in comparison to the cited papers and the scores obtained [5,28].

## 4. Conclusions

In conclusion, we have shown here that the formulation of an isolipidic shortbread cookie recipe that reduces the butter amount (i.e., about 11% and 3% less butter than the total butter amount of the classic shortbread cookie for HSc10% and HSc5%, respectively) and replaces it with the fat naturally present in food waste hazelnut skin of the Romana variety helps to reduce the saturated fat in favor of monounsaturated fats. Although an amount of trans-fatty acid persists, they are still within the limit suggested by WHO [9]. The use of a different procedure to separate hazelnut skin from the kernel, instead of roasting, will probably help to reduce the amount of trans-fatty acid. Among the two percentages of Romana hazelnut skin included, only the 10% incorporation determined a significant increase in the number of monounsaturated fats. The sensory analysis revealed higher consumer acceptance of the HSc5% formulation, with scores comparable to the control. Although a lower consumer acceptance index for the HSc10% formulation was found, it was greater than other hazelnut-skin-enriched products and with the highest percentage of hazelnut skin used. Moreover, the purchase intention analysis revealed that 35% of the interviewed population would buy the HSc10% formulation, indicating that this product, beyond the ethical dimension of using a food waste matrix in support of the circular economy, can attract the commercial interest of part of the population. Antioxidant and fiber content analyses are necessary and they are planned as future goals. Moreover, the replacement of part of the butter with an unsaturated vegetable fat in the recipe could be a solution to further improve the saturated/unsaturated ratio.

## Figures and Tables

**Table 1:** HS proximate composition (g/100 g dry weight).

	Moisture	Protein ^1^	Fat	Carbohydrates ^2^	Ash	kcal/100 g ^3^	kJ/100 g ^3^
HSR	6.31 ± 0.02 ^a^	9.70 ± 0.14 ^a^	26.71 ± 0.04 ^b^	60.41	3.19 ± 0.02 ^a^	487.91	2041.43
HSG	3.13 ± 0.02 ^d^	9.57 ± 0.09 ^b^	28.98 ± 0.12 ^a^	58.59	2.85 ± 0.04 ^b^	516.78	2101.09
HSL	3.44 ± 0.05 ^c^	6.94 ± 0.03 ^d^	26.30 ± 0.14 ^c^	63.90	2.86 ± 0.03 ^c^	502.17	2161.20
HST	5.83 ± 0.04 ^b^	7.90 ± 0.06 ^c^	20.36 ± 0.17 ^d^	69.49	2.25 ± 0.04 ^d^	464.07	1941.67

Data are means ± standard deviation of two (*n* = 2) replicates. Different letters indicate significant differences (*p* ≤ 0.05) according to one-way analysis of variance. Lowercase letters represent Fisher’s comparison test among different cultivars. HSR: hazelnut skin, Tonda Gentile Romana; HSG: hazelnut skin, Tonda di Giffoni; HSL: hazelnut skin, Tonda Gentile delle Langhe; HST: hazelnut skin, Turkish Tombul (HST). ^1^ Conversion factor: 6.25. ^2^ As difference (i.e., 100 − (g [protein + fat + ash] in 100 g of dry weight sample)). ^3^ 100 g of edible part.

**Table 2:** Ingredients of the two experimental cookie recipes and the control.

	CSc	HSc5%	HSc10%
flour	48.3%	43.9%	40.5%
HSR	-	5%	10%
butter	20.2%	19.6%	18%
sugar	19%	19%	19%
eggs	11.6%	11.6%	11.6%
vanillin	0.1%	0.1%	0.1%
baking powder	0.8%	0.8%	0.8%

CSc: control cookie; HSc5%: hazelnut skin cookie 5%; HSc10%: hazelnut skin cookie 10%; HSR: Romana hazelnut skin.

**Table 3:** Experimental cookie proximate compositions (g/100 g dry weight).

	Moisture	Protein ^1^	Fat	Carbohydrates ^2^	Ash	kcal/100 g ^3^	kJ/100 g ^3^
CSc	4.79 ± 0.06 ^b^	7.86 ± 0.06 ^a^	21.09 ± 0.21 ^a^	70.50	1.14 ± 0.02 ^b^	474.11	1983.69
HSc5%	3.18 ± 0.34 ^c^	7.96 ± 0.29 ^a^	21.44 ± 0.67 ^a^	69.21	1.39 ± 0.02 ^a^	485.72	2032.24
HSc10%	5.71 ± 0.04 ^a^	7.34 ± 0.23 ^b^	21.31 ± 0.46 ^a^	69.97	1.39 ± 0.01 ^a^	472.40	1976.54

Data are means ± standard deviation of two (*n* = 2) replicates. Different letters indicate significant differences (*p* ≤ 0.05) according to one-way analysis of variance. Lowercase letters represent Fisher’s comparison test among different experimental cookies. CSc: control cookie; HSc5%: hazelnut skin cookie 5%; HSc10%: hazelnut skin cookie 10%; HSR: Romana hazelnut skin. ^1^ Conversion factor: 6.25. ^2^ As difference (i.e., 100 − (g [protein + fat + ash] in 100 g of dry weight sample)). ^3^ 100 g of edible part.

**Table 4:** Fatty acid content of HSR and experimental cookies (%).

	HSR	CSc	HSc5%	HSc10%
Caprylic acid, C8:0	n.d.	0.35 ± 0.07	0.30 ± 0.14	n.d.
Capric acid, C10:0	n.d.	1.40 ± 0.71	2.10 ± 0.14	1.85 ± 0.21
Undecanoic acid, C11:0	n.d.	n.d.	0.45 ± 0.07	0.55 ± 0.07
Lauric acid, C12:0	n.d.	2.95 ± 0.50	3.35 ± 0.21	3.45 ± 0.07
Tridecanoic acid, C13:0	n.d.	n.d.	0.15 ± 0.07	0.25 ± 0.07
Myristic acid, C14:0	n.d.	12.15 ± 0.07 ^b^	12.75 ± 0.07 ^a^	12.15 ± 0.21 ^b^
Pentadecanoic acid, C15:0	n.d.	0.85 ± 0.07	0.85 ± 0.07	0.90 ± 0.01
Palmitic acid, C16:0	6.60 ± 0.57 ^c^	40.05 ± 3.04 ^a^	39.10 ± 1.13 ^a^	33.75 ± 0.64 ^b^
Palmitoleic acid, C16:1n7	n.d.	0.95 ± 0.07	1.05 ± 0.07	1.10 ± 0.14
Stearic acid, C18:0	1.85 ± 0.07 ^b^	8.20 ± 0.01 ^a^	7.25 ± 0.07 ^a^	5.90 ± 1.14 ^a^
Oleic acid, C18:1n9	80.50 ± 0.14 ^a^	28.65 ± 1.48 ^c^	28.00 ± 0.28 ^c^	34.35 ± 1.20 ^b^
Linolelaidic acid, C18:2 n6,9 all trans	11.05 ± 0.49 ^a^	4.45 ± 0.50 ^c^	4.60 ± 0.14 ^bc^	5.75 ± 0.49 ^b^
Total saturated FA (SFA)	8.50	66.00	66.30	58.80
Total unsaturated FA (UFA)	80.50	29.60	29.05	35.45
UFA/SFA	9.53	0.45	0.44	0.60
UFA/TFA	7.29	6.65	6.32	6.17

Data are means ± SD (*n* = 2). Means with different letters within a row are significantly different (*p* < 0.05) according to one-way analysis of variance. Lowercase letters represent Fisher’s comparison test among different experimental cookies. CSc: control cookie; HSc5%: hazelnut skin cookie 5%; HSc10%: hazelnut skin cookie 10%; HSR: Romana hazelnut skin. n.d.: not detected. SFA: saturated fatty acids; UFA: unsaturated fatty acids; TFA: trans-fatty acids.

**Table 5:** Acceptance means for the sensory test of the three cookie samples.

Acceptance Means		Cookies	
	CSc	HSc5%	HSc10%
Overall liking	7.68 ± 1.61 ^a^	7.31 ± 1.62 ^b^	6.20 ± 1.58 ^c^
Flavor	7.39 ± 1.63 ^a^	7.29 ± 1.63 ^a^	6.40 ± 1.60 ^b^
Aroma	7.80 ± 1.75 ^a^	7.59 ± 1.76 ^b^	6.03 ± 1.72 ^c^
Aroma persistence	7.46 ± 1.85 ^a^	7.41 ± 1.84 ^a^	5.85 ± 1.81 ^b^

CSc: control cookie; HSc5%: hazelnut skin cookie 5%; HSc10%: hazelnut skin cookie 10%. Data are means ± SD (*n* = 118). Means followed by different letters indicate significant differences (*p* ≤ 0.05) according to one-way analysis of variance. Lowercase letters represent Fisher’s comparison test among different experimental cookies.

## Data Availability

The datasets generated for this study are available on request to the corresponding author.
